# Time-Restricted Eating Alters Food Intake Patterns, as Prospectively Documented by a Smartphone Application

**DOI:** 10.3390/nu12113396

**Published:** 2020-11-05

**Authors:** Samar Malaeb, Tasma Harindhanavudhi, Katrina Dietsche, Nick Esch, Emily N. C. Manoogian, Satchidananda Panda, Douglas G. Mashek, Qi Wang, Lisa S. Chow

**Affiliations:** 1 Division of Diabetes, Endocrinology and Metabolism, Department of Medicine, University of Minnesota, Minneapolis, MN 55455, USA; hari0049@umn.edu (T.H.); dmashek@umn.edu (D.G.M.); chow0007@umn.edu (L.S.C.); 2 College of Biological Sciences, University of Minnesota, Minneapolis, MN 55455, USA; diets018@umn.edu (K.D.); eschx037@umn.edu (N.E.); 3 Salk Institute for Biological Sciences, La Jolla, CA 92037, USA; emanoogian@salk.edu (E.N.C.M.); panda@salk.edu (S.P.); 4 Department of Biochemistry, Molecular Biology and Biophysics, University of Minnesota, Minneapolis, MN 55455, USA; 5 Clinical and Translational Science Institute, University of Minnesota, Minneapolis, MN 55455, USA; wangx890@umn.edu

**Keywords:** time-restricted eating, eating occasions, dietary patterns

## Abstract

Time-restricted eating (TRE) can facilitate weight loss, yet its effect on eating patterns remains unknown. Twenty adults with BMI ≥ 25 kg/m^2^ underwent a 12-week randomized trial, examining the effect of an 8-h, time-restricted eating intervention on dietary patterns. Oral intake was documented using a smartphone. Dietary patterns, assessed as frequency of eating occasions (EOs) and types of meals/snacks and beverages, were compared between baseline (T0), early-intervention (T1), and end-intervention (T2). At T1 and T2, both groups had less EOs compared to T0, with greater reduction seen in the TRE group (−28%) than the non-TRE group (−12%) at T2 (*p* = 0.01 vs. non-TRE). Comparing T1 to T0, the TRE group documented less incomplete meals (−32.5%: *p* = 0.02), high quality snacks (−23.6%: *p* = 0.03), and low quality snacks (−36.6%: *p* = 0.004). Comparing T2 to T0, the TRE group documented less incomplete meals (−33.9%: *p* = 0.03), high quality snacks (−28.1%: *p* < 0.001) and low quality snacks (−51.2%: *p* < 0.001). Caffeinated beverage intake was reduced in the TRE group at T1 (−20.2%) and T2 (−28.8%) vs. T0, but remained unaltered in the non-TRE group. By using a smartphone application to document dietary intake, TRE significantly reduced the number of EOs, snacks, and caffeinated beverages, relative to baseline and relative to the non-TRE.

## 1. Introduction

The prevalence of obesity in the United States and around the world is rising, with the latest reports showing that 20.9% of adolescents and 42.4% of US adults were classified with obesity in 2017–2018 [1]. Given the associated morbidity and mortality from obesity and the substantial socioeconomic burden [2,3], there is an intense medical and public interest in dietary and lifestyle management to mitigate obesity and its associated complications.

Of the various dietary patterns that are recommended for weight loss, time-restricted eating (TRE) gained significant interest within recent years. TRE is a daily eating pattern in which all nutrient intake occurs within a specific period (typically within 8–12 h) daily, with no intentional attempt to alter nutrient quality or quantity [4,5]. TRE facilitates weight loss, similar to daily calorie restriction [6]. In addition, earlier animal studies and human trials showed that TRE appears to protect against the adverse metabolic effects of high-fat diets [7,8] and promotes a broad spectrum of health benefits, particularly metabolic and cardiovascular disease [9,10,11,12]. Previous randomized controlled trials examined TRE interventions in various populations, showing significant weight loss and improvement of metabolic parameters, along with a reduction of caloric intake [12,13,14]. While other studies demonstrated that TRE intervention improved cardiometabolic parameters independent of weight loss [9,15,16], it remains unclear whether the benefit of TRE is explained by a reduction of overall caloric intake or alteration of dietary patterns. 

Historically, paper-based food records were the method of choice to assess dietary intake. Paper-based food records, however, are inconvenient due to the need to carry/maintain the record during the documentation period. Other options for capturing food intake are the 24-h dietary recall and 7-days food records [17]. However, this option is dependent on participant recall and might be incomplete due to its retrospective nature [18,19]. Recently, technology-based mobile app-based food records have emerged. Previous studies demonstrated that the accuracy of food records and calculated energy intake were comparable between either an online computer or smartphone-based food records, and paper-based records [20,21] or weighed food intake/waste method [22]. Moreover, technology-based mobile food records facilitate and improve self-monitoring adherence and weight loss [23,24], as they can capture food records prospectively rather than retrospectively. Previous studies examining TRE intervention analyzed data on caloric intake or food patterns, using either paper-based food records or dietitian interviews [13,14,15,25]. The use of a smartphone application was recently incorporated into TRE intervention studies [11,16,26,27]. Recent studies demonstrated that the use of smartphone application that centered on food pictures with a textual option to track food records, without an attempt to change dietary quantity or quality, might reduce caloric intake and subsequently promote weight loss in healthy individuals with overweight [26]; improve metabolic parameters and body composition in individuals with metabolic syndrome [11]; and reduce eating occasions in healthy individuals with overweight status [27].

The extent to which TRE intervention alters dietary quality and composition, as captured by a prospective mobile food record, remains unknown. Therefore, we examined TRE intervention-related alteration of eating patterns over a 12-week period, using a smartphone-based application centered on food pictures with textual option documentation, in metabolically healthy adults with overweight or obesity (BMI ≥ 25 kg/m^2^). We previously reported that TRE reduced eating occasions [27]. The novelty of the current study was that it examined the effect of TRE on dietary patterns. We hypothesized that prospective food tracking using smartphone application alters dietary patterns, with a specific reduction of meal and snack frequency with TRE. 

## 2. Materials and Methods

This study analyzed dietary data patterns from the “See Food” study, which was a randomized, unblinded, interventional feasibility clinical trial conducted at the University of Minnesota (Minneapolis, MN) between October 2017 and December 2018, to evaluate the effects of TRE on body composition and metabolic outcomes in adults with overweight or obesity, for 12 weeks. This is a companion study to the original findings, where the study design, protocol, and primary endpoint analyses were previously published [27]. In brief, a total of 47 adult participants aged 18–65 years with a BMI ≥ 25 kg/m^2^ who had stable sleep and work schedule, and owned a smartphone were enrolled. Twenty participants completed the study and had available data for analysis. All subjects gave their informed consent for inclusion before they participated in the study. The study was conducted in accordance with the Declaration of Helsinki, and the protocol was approved by the Institutional Review Board of the University of Minnesota (Project identification code number: 1701M06001) on 21 March 2017. ClinicalTrial.gov identification number is NCT03129581.

### 2.1. Study Design/Intervention

Potential participants were asked to document the timing of their food intake, using a smartphone application (My Circadian Clock: mCC) on their smartphones, for at least 1 week, to determine their eating window and ability to log dietary intake. Participants who demonstrated an eating window of 14 h or greater were enrolled and randomized into one of two groups—an intervention group (TRE, or time-restricted eating) or a non-TRE group (unrestricted eating). The participants in the TRE group were counseled to self-select and maintain a daily 8-h eating window during the 12-week intervention while tracking all oral intake using the mCC application. The participants were permitted unrestricted eating during this eating window and were specifically not provided instruction on altering their dietary patterns or dietary intake during this eating window. The participants in the non-TRE group were instructed to eat as per their usual habits, while tracking all oral intake using the mCC application. 

### 2.2. “myCircadianClock” Smartphone Application (mCC)

The “myCircadianClock” smartphone application is freely available for download for iPhone or Android platforms and allows a participant to use the smartphone camera function to take a picture of the ingested food or beverage whenever they eat, without required food quantification. In addition, participants manually entered the description of the food and beverage in the mCC app. The timestamp and the location were noted and transferred while de-identified, to a cloud-based data server. The utility and validity of this application in free-living humans ererecently published [26]. The study team remotely monitored data from the mCC app on a weekly basis to ensure data fidelity, adequate logging, and for the TRE group, compliance with the eating window. Participants were notified by either phone call, email, or text about their eating-window compliance in the TRE group and logging adherence in both the TRE and non-TRE groups. Participants were considered adherent to logging if they documented ≥2 meals ≥5 h apart >1 day/week. Participants were considered adherent to the TRE intervention if all entries were entered within 15 min of their designated eating window. Other than establishing a consistent daily 8-h eating window, participants were not provided further instruction on diet intake, diet quality, or energy intake.

### 2.3. Outcome Measurements

Dietary patterns were determined over 14-day periods at baseline (T0: just prior to randomization), immediately after randomization (early intervention: T1), and at the end of study intervention (end-intervention: T2). Two authors (KD, NE) independently reviewed the food record data from the mCC app, to characterize eating occasions (EO) and classify food intake (detailed below). If discrepancies existed between the primary reviewers (KD, NE) about the EO and food-based classification, the results were adjudicated by consensus review from a third party (SM and TH).

***Eating Occasions (EO):*** In this study, the neutral term “eating occasion”, was used to describe any occasion where any food or beverage (other than water or medicine) was ingested. An EO was considered distinct if it occurred >15 min apart from another EO [17]. 

***Food-Based Classification:*** To characterize the type of EO, and thus get a better understanding of dietary pattern, a food-based classification scheme was used, based on the work of Lennernas & Andersson [28]. This method classified food consumed into categories, based on nutritional profile (e.g., plant vs. animal, nutrient density, energy density, etc.). Based on this classification, six EO types were defined, ranging from a complete meal (high nutrient density; contains both animal and plant foods) to a low-quality snack (low nutrient density) [28] (Table 1). Supplementary Table S1 describes the detailed classification of each EO type.

***Beverages:*** Beverages were classified separately by number and type, using image and participant descriptions, which were then organized into the following categories—sugary, artificial sweetened, dairy, caffeinated, alcoholic beverages, and water.

### 2.4. Statistical Analysis

A linear mixed model was used to evaluate the change in eating occasions, food, and beverages classification from pre- to post-intervention between groups. Models included a random intercept to account for correlations among repeated measures within-subject. Results are reported as mean and standard error. Pearson correlation coefficient was calculated by treatment group to evaluate the correlation between weight loss and change in dietary patterns. Statistical analyses were performed in SAS 9.4 (SAS Institute Inc., Cary, NC, USA). A *p*-value ≤ 0.05 was considered to be statistically significant. Given the pilot nature of the study, no adjustments to *p*-values were made.

## 3. Results

A total of 20 participants completed the study (11 in the TRE group and 9 in the non-TRE group). Baseline characteristics of randomized participants were previously reported [27]. In brief, the mean age of participants was 45.5 ± 12.1 years, 85% were female, and mean BMI was 34.1 ± 7.5 kg/m^2^, and the majority of participants were non-Hispanic white [ethnicity (3 Hispanic/Latino, 17 non- Hispanic/Latino) and race (3 black, 17 white)] (Supplementary Table S2). There were no statistically significant differences in baseline characteristics except for higher triglycerides, TSH (thyroid stimulating hormone), diastolic blood pressure, and 2-h oral glucose tolerance test (OGTT) values in the TRE group than the non-TRE group (*p* < 0.05) (Supplementary Table S2). As previously reported, the TRE group narrowed the eating window (pre 15.2 ± 0.7 vs. post 9.9 ± 2.0 h; *p* < 0.01), whereas the eating window of the non-TRE group remain unchanged (pre 15.5 ± 1.1 vs. post 15.1 ± 1.1 h; *p* = 0.24) [27]. As previously reported, mCC logging adherence was ~92% adherence, prior to randomization and ~80–90% adherence during the 12-week intervention [27]. 

### 3.1. Change in Eating Occasions (EO) over Time 

In this present analysis, we examined the temporal change in EO overtime at immediate-intervention (T1) and at the end intervention (T2), and specifically examined changes in dietary patterns (Table 2). Regardless of the intervention group, over the 14 days immediately after randomization (T1), both TRE and non-TRE groups significantly reduced EO per day by 14%, compared to baseline (T0) (*p* = 0.0009 for TRE and *p* = 0.0018 for non-TRE groups). When assessing the diet pattern over 14 days just prior to end-intervention (T2), both the TRE and non-TRE groups had significantly less EO per day than baseline, with a greater reduction in the TRE group (−28%) compared to the non-TRE group (−12%) at end-intervention (*p* = 0.01 compared with non-TRE). 

### 3.2. Changes of Dietary Patterns over Time

Both the TRE and non-TRE groups changed their dietary pattern over the course of the study. At baseline (T0), there was no difference in the types of meals between the TRE and non-TRE groups. The TRE group specifically consumed less incomplete meals, and high and low-quality snacks at T1 and T2, compared to T0. Comparing T1 to T0, the TRE group consumed less incomplete meals (−32.9%, *p* = 0.02), high quality snacks (−23.6%, *p* = 0.03), and low quality snacks (−36.6%, *p* = 0.004). Comparing T2 to T0, the TRE group continued to consume less incomplete meals (−33.9%: *p* = 0.03), high quality snacks (−28.1%, *p* < 0.0001), and low quality snacks (−51.2%, *p* < 0.001) (Table 2). In response to the TRE intervention, the TRE group significantly reduced intake of high-quality snacks from T2 to T0 (−49.9% vs. −6.3%, *p* = 0.008) and from T2 to T1 by (−34.4% vs. 13.6%, *p* = 0.03), as compared to the non-TRE group. 

In the non-TRE group, participants had less specific dietary pattern changes over the study period. The non-TRE group documented a less complete meal by 27.1% at early intervention (T1) (*p* = 0.005), which became non-significant at the end-intervention (T2) (*p* = 0.35 compared to baseline). The non-TRE group consistently had less vegetarian meals by at T1 (−58.3%, *p* = 0.005) and T2 (48.6%, *p* = 0.02), when compared to baseline. Similar to the TRE group, the non-TRE group documented less low-quality snacks at both T1 (−18.9%, *p* = 0.03) and T2 (−22.6%, *p* = 0.01), when compared to baseline (Table 2). The non-TRE, but not the TRE groups, also had significantly less mixed quality snacks (−50.0%, *p* < 0.001) from T2 to baseline.

### 3.3. Alteration in Beverage Documentation over Time

We examined the alternation in beverage documentation over time. In both interventions, the frequency of water intake declined by 50.2% in the TRE group, and by 65% in the non-TRE group, which was not statistically different between groups (Table 3). Both the TRE and non-TRE groups documented less dairy beverages by −73.7% (*p* = 0.02) and 35.5% (*p* = 0.04), respectively, from T2 to baseline (Table 3). Interestingly, the frequency of documenting sugary and artificially sweetened beverages during the study did not change in either group. Specific to TRE, the TRE group significantly reduced caffeinated beverages (−20.2%, *p* = 0.003) at T1 and T2 (−43.2%, *p* < 0.0001), relative to baseline. 

### 3.4. Correlation between Alteration in Dietary Patterns and Weight Loss

We also examined if the reduction in EO correlated with weight loss; no correlation was observed in either group (*p* = 0.98 for the TRE group, *p* = 0.48 for the non-TRE group). An increase in high quality snacks was positively correlated with percent weight loss in the non-TRE group (r = 0.670, *p* = 0.05), while an increase in mixed quality snacks was negatively correlated with percent (r = −0.602, *p* = 0.05) and absolute weight loss (r = −0.595, *p* = 0.05), respectively in the TRE group. No significant correlations were observed between other food types/beverages and weight loss (Supplementary Table S3).

## 4. Discussion

The purpose of this study was to examine the effects of a TRE on dietary patterns, in addition to the potential impact of food tracking using mobile food records. We found that TRE (with no specified food intake during the eating window) reduced the number of eating occasions and specifically resulted in the consumption of fewer snacks and caffeinated beverages. The study also demonstrated the impact of food tracking on alteration of dietary patterns in non-TRE participants, showing that tracking of food intake might have reduced eating occasions, although this did not translate into weight loss.

Previously published results of this same study showed that TRE improved metabolic parameters and promoted weight loss in individuals with overweight or obesity [27]. Our current analysis adds to this literature by examining the effects of smartphone-based tracking on eating occasions/dietary intake and whether the imposition of eating windows might further alter eating occasions/dietary intake. Several previous TRE studies showed that TRE reduced eating events across a wide swath of participants, ranging from healthy overweight adults to adults with metabolic syndrome [11,26,27]. In this study, we reported that reduction in eating occasions was immediately seen after randomization, regardless of the intervention arm. At the end of the 12 weeks intervention, eating occasions continued to decline in the TRE group, while eating occasions in the non-TRE group remained unchanged. Taken together, this temporal trend of dietary patterns indicated (1) the practicability and sustainability of 12-week duration of TRE in a real-life setting; and (2) the effect of self-dietary monitoring in the non-TRE participants. While this present study did not quantitatively assess caloric intake, we observed weight loss in the TRE group without any alteration in sleep or physical activity, suggesting that caloric intake might be reduced despite unrestricted food intake within the window [27]. 

Eating patterns in humans lack a structured 3 meals per day pattern but are rather highly variable throughout a 24-h period [26], resulting in the challenges in characterizing dietary intake patterns. Several approaches were used to operationally define meals and snacks [17]. In this study, we applied the food-based classification of eating events (FBCE) developed by Lennernas et al. [28] to define eating patterns that reflect qualitative categorization within dietary composition. This method classifies each eating occasion from food recall into meals and snacks, based on visible properties (food types) and invisible properties (nutrients) [28], and is designed to reflect food combinations without considering the amounts. Therefore, we applied the FBCE method to the data from myCircadianClock (mCC) smartphone application [26] during TRE intervention, unlike previous TRE studies that assessed dietary history using self-dietary records or dietitian interviewing to categorize dietary intake into major macronutrient composition [13,14,15,16]. Granted, the lack of quantifying calories and food volume is a limitation of this study. However, by studying the same individual over time, we are able to report dietary alterations over time, associated with the use of real-time mobile food records, which had more real-world applicability.

Beyond reducing EOs, TRE altered dietary composition. Changes in the reduction of “snacks” over time suggest that the effect of TRE on the frequency of eating occasions might be due to a reduction in snack intake as well as alteration of snack quality. Therefore, it could imply that TRE might influence the shift in eating patterns to focus on “meal” consumption, characterized by a combination of more than one food category rather than “snack” consumption characterized by a single food component with high-fat and low-nutrient content. Our findings raise basic questions about the significance of eating patterns on health—whether certain patterns (eating more/less frequent meals or more/less frequent snacks) are associated with health benefits, and whether alterations in these eating patterns might affect health. An extensive review by Bellisle, in 2014 [29], reported inconsistent and often divergent relationships between snacking and health, particularly the effect on body weight. Certainly, variability in defining meals vs. snacks, snack food types, outcome measures, and study populations, play a significant role in the discrepant outcomes [30]. Yet, nutrient-poor, high-energy density snacks are “unquestionably deleterious”, and are a major contributor to the obesity epidemic. Simply put, snack quality matters [29,30]. Similarly, our study distinguished snacks by quality and showed that an increase in high-quality snacks was positively correlated with weight loss in the non-TRE group, while an increase in mixed quality snacks was negatively correlated with weight loss in the TRE group. Therefore, our study provided novel insights into the TRE-induced changes in meal and snack quality, beyond the observed reduction in eating occasions [27]. In addition, our observation that TRE results in less consumption of caffeinated products is consistent with previous findings from Gill and Panda [26], that humans tend to consume a specific type of food in a time-dependent manner. For example, coffee is typically consumed in the morning. Thus, delaying AM eating to accommodate the TRE imposed 8-h eating window could explain the reduction of caffeinated beverage consumption.

The study had several strengths. In particular, the study was novel in using a prospective, technology-based mobile food record (mCC application) to categorize eating occasions. The study captured the effect of a randomized TRE intervention on dietary patterns. This study was different from previous TRE interventions that assessed dietary history using paper-based (check) records or recall dietitian interviewing [13,14,15,16]. Although we recognize challenges in the complexity of defining eating patterns, particularly eating occasions, nutrient intakes, and diet quality [17], we believe that food-based classification of the eating events (FBCE) classification system offers the advantage of capability, to capture what constitutes a meal vs. a snack, as well as an idea of meal quality and patterning of eating events [17]. We were able to utilize longitudinal data to examine the temporal changes in dietary patterns.

Several limitations of this study are noted. First, we acknowledge the important limitation that the mCC app was used for both diet assessment and diet intervention. We recognized that more objective data would be obtained if assessment tools independent of the intervention were used. Indeed, the non-TRE group altered their dietary composition in response to the mCC app usage. Yet, simply tracking dietary intake is well-known to modify dietary intake [31], which was consistent with our findings. By comparing against a non-TRE control group as well as baseline findings, we find that the observed changes in the TRE group are valid. Yet for future TRE-based studies, we would recommend the inclusion of dietary intake/dietary quality assessments, independent of the mCC app, such as the 24-h dietary recall [32,33] for more objective findings. Another limitation is that, since the assessment of dietary composition was not the primary objective of the original study, this study did not measure food intake by dietitian interviews or using validated food recalls/questionnaires. Additionally, there were various criteria for time separation between eating occasions, ranging from 15 min to 1 h of different studies used previously. Likewise, there is a variation amongst studies including vs. excluding beverages from eating occasions. Although we used a novel app-based and user-friendly approach in assessing dietary intake, we acknowledge that our study could not ascertain food volume or energy intake quantification from the mCC-based images. This limitation confounds the accuracy of actual caloric intake and limits the comparison with previous studies, to only an assumption of a reduction of energy intake, as a result of a reduction of eating occasions. We also acknowledge that capturing the diet patterns for 14 days at baseline, early intervention, and end-intervention, might not reflect ongoing diet fluctuations during the intervention. Furthermore, our study population consisted of healthy participants, BMI ≥ 25 kg/m^2^, thus, we acknowledge that these findings might not be generalizable to other populations. Further studies on dietary self-monitoring during TRE could focus on different methods in assessing meal patterns that not only focus on eating frequency but also collect contextual information that influences eating patterns and diet quality, such as time, location, and activities while eating. Finally, it is important to note that food recording using the app does not necessarily prove actual consumption, but this can be said as a limitation to all forms of diet reporting or recall. We acknowledge this limitation and address it by inferring general “patterns of eating” from the subjects’ records, and favoring the term “documented consumption” over “consumed”.

## 5. Conclusions

Using a smartphone-based application for self-dietary monitoring, we found temporal changes in the reduction of eating occasions in both the TRE and the non-TRE groups. With the 8-h TRE intervention, the most prominent pattern was a reduction of snacks enriched in high fat-dense, low nutrient-dense (sugar and fat), and high nutrient-dense foods (animal and starch/grain product), as well as a reduction of caffeinated beverages.

## Figures and Tables

**Table 1:** Food-Based Classification of Eating Events (modified from Lennernas et al. [28]).

Complete Meal	CM	A meal containing an animal product, a grain/starch, and a fruit/vegetable
Incomplete Meal	IM	A meal containing an animal product and a grain/starch
Less-Balanced Meal	LM	A meal containing an animal product and a fruit/vegetable
Vegetarian Meal	VM	A meal containing a grain/starch and a fruit/vegetable
High-Quality Snack	HS	Nutrient-dense foods eaten individually, i.e., meat or grain/starch or fruits/vegetables.
Low-Quality Snack	LS	High-fat and/or low-nutrient foods, (i.e., highly refined and processed food products) eaten individually
Mixed-Quality Snack	MS	Combination of HS + LS

**Table 2:** Frequency of food types T0, T1, and T2 in the TRE and the non-TRE groups.

	TRE (*N* = 11)					Non-TRE (*N* = 9)				
Food Type (events/day)	T0	T1	*p*-Value T1 vs. T0	T2	*p*-Value T2 vs. T0	T0	T1	*p*-Value T1 vs. T0	T2	*p*-Value T2 vs. T0
Overall eating occasion *	5.4 ± 0.4	4.6 ± 0.4	*p* < 0.001	3.8 ± 0.4	*p* < 0.001	5.6 ± 0.5	4.9 ± 0.4	0.002	4.9 ± 0.4	0.007
Complete meal **	1.0 ± 0.2	1.1 ± 0.2	0.47	0.9 ± 0.2	0.07	1.1 ± 0.2	0.8 ± 0.2	0.005	1.0 ± 0.5	0.35
Incomplete meal	0.5 ± 0.1	0.3 ± 0.1	0.02	0.3 ± 0.1	0.03	0.5 ± 0.1	0.5 ± 0.1	0.92	0.4 ± 0.1	0.84
Less balanced meal	0.4 ± 0.1	0.4 ± 0.1	0.84	0.5 ± 0.1	0.08	0.3 ± 0.1	0.3 ± 0.1	0.81	0.2 ± 0.1	0.51
Vegetarian meal	0.1 ± 0.05	0.1 ± 0.05	0.28	0.1 ± 0.05	0.99	0.2 ± 0.1	0.1 ± 0.1	0.005	0.1 ± 0.05	0.02
High quality snack ***	0.9 ± 0.1	0.7 ± 0.1	0.03	0.5 ± 0.1	*p* < 0.001	0.8 ± 0.2	0.7 ± 0.2	0.18	0.8 ± 0.2	0.63
Mixed quality snack ****	0.3 ± 0.1	0.3 ± 0.1	0.32	0.2 ± 0.1	0.63	0.6 ± 0.1	0.5 ± 0.1	0.19	0.3 ± 0.1	*p* < 0.001
Low quality snack	0.9 ± 0.2	0.5 ± 0.2	0.004	0.4 ± 0.2	*p* < 0.001	1.3 ± 0.2	1.1 ± 0.2	0.03	1.0 ± 0.2	0.01

T0: Baseline, T1: Early Intervention, T2: End-intervention * *p* = 0.01 comparison of T2 and T0 and T2 and T1 between the TRE and the non-TRE group. ** *p* = 0.01 comparison of T1 and T0 between the TRE and the non-TRE group; *p* = 0.002 comparison of T2 and T1 between the TRE and the non-TRE group. *** *p* = 0.008 comparison of T2 and T0 between the TRE and the non-TRE group; *p* = 0.027 comparison of T2 and T1 between the TRE and the non-TRE group. **** *p* = 0.014 comparison of T2 and T0 between the TRE and the non-TRE group.

**Table 3:** Frequency of beverages types at T0, T1, and T2 in the TRE and non-TRE groups.

	TRE (*N* = 11)					Non-TRE (*N* = 9)				
Beverages	T0	T1	*p*-Value T1 vs. T0	T2	*p*-Value T2 vs. T0	T0	T1	*p*-Value T1 vs. T0	T2	*p*-Value T2 vs. T0
Water	1.9 ± 0.4	1.5 ± 0.4	0.01	0.9 ± 0.4	<0.0001	2.1 ± 0.5	1.3 ± 0.5	<0.0001	0.7 ± 0.5	<0.0001
Caffeinated *	1.4 ± 0.1	1.1 ± 0.1	0.003	0.8 ± 0.1	<0.0001	0.8 ± 0.2	0.7 ± 0.2	0.11	0.9 ± 0.2	0.93
Sugary	0.1 ± 0.1	0.1 ± 0.1	1.0	0.1 ± 0.1	0.91	0.3 ± 0.1	0.3 ± 0.1	0.72	0.3 ± 0.1	0.63
Artificially sweetened	0.03 ± 0.02	0.03 ± 0.02	0.72	0.01 ± 0.02	0.16	0.04 ± 0.02	0.03 ± 0.02	1.0	0.05 ± 0.02	0.44
Dairy	0.1 ± 0.1	0.1 ± 0.1	0.06	0.03 ± 0.1	0.02	0.3 ± 0.1	0.2 ± 0.1	0.33	0.2 ± 0.1	0.04
Alcohol **	0.6 ± 0.2	0.7 ± 0.2	0.32	0.5 ± 0.2	0.23	0.4 ± 0.2	0.2 ± 0.2	0.24	0.3 ± 0.2	0.93

T0: Baseline, T1: Early Intervention, T2: End-intervention * *p* < 0.0001 comparison of T2 and T0 between the TRE and the non-TRE group, *p* = 0.0006 comparisons of T2 and T1 between the TRE and the non-TRE group. ** *p* = 0.026 comparison of T2 and T1 between the TRE and the non-TRE group.

## Supplementary Materials

The following are available online at https://www.mdpi.com/2072-6643/12/11/3396/s1. Table S1 Title: Food categories and their nutrient properties as the base for categorization of Eos. Table S2 Title: Baseline Characteristics of study participants. Table S3 Title: Correlation between weight loss and change in eating occasion, food types, and beverages.
