# Supplementary material for: Time-Restricted Eating Alters Food Intake Patterns, as Prospectively Documented by a Smartphone Application
Source: Nutrients. 2020 Nov 5;12(11):3396. doi: 10.3390/nu12113396 (PMC7694329; doi:10.3390/nu12113396)
Supplement: Supplementary file 1 [file nutrients-12-03396-s001.pdf]

Supplemental Table S1: Food categories and their nutrient properties as the base for categorization of eating occasions

**Category**	**Origin**	**Food Examples**	***Nutrient/Energy density***	**Nutrients**
Category a	Animal origin	Meat and meat products, fish and shellfish, poultry, egg, milk and cheese	*High nutrient density*	Animal protein and fat, iron, zinc, calcium
Category b	Plant Origin	Rice, pasta, bread, dried legumes, seeds, potatoes	*High nutrient density*	Starch, plant protein, dietary fibre
Category c	Plant origin	Green vegetables, fruit, berries, roots	*High nutrient density, low energy density*	Starch, carontenoids, ascorbic acid
Category d	Plant origin	Nuts, olives, avocado	*High fat density*	Plant fat, plant protein
Category e	Animal and plant origin	Cooking fat, spreads, cream, fatty sauces	*High fat density*	Fat
Category f	Plant origin	Products in which white sugar often is added, beverages containing alcohol, ice cream, sweets, chocolate, biscuits, sweet desserts	*Low nutrient density*	Sugar, fat, alcohol
Category g	---	Water, coffee, tea, unsweetened light beverages	*No energy*	No nutrients
		Categorization of EOs due to the combination of food categories		
Meal or snack	Abbreviation	Category of meal/snack	Combination	Example
Meals	CM	Complete meal	a+b+c	Meat, potatoes or bread, carrots
	IM	Incomplete meal	a+b	Meat, potatoes or bread
	LM	Less balanced meal	a+c	Meat, carrots
	VM	Vegetarian meal	b+c	Potatoes or bread, carrots
Snacks	HS	High-quality snack	a or b or c	An apple
	MS	Mixed-quality snack	Any of a or b or c and/or d and/or e and/or f	An apple and Some chocolate
	LS	No-quality snack	e and/or f	Some chocolate
	NC	No-energy snack	g	Coca cola light

Supplemental Table S2: Baseline characteristics of study participants [a]

**Characteristic** [b]	**TRE (*n* = 11)**	**non TRE (*n* = 9)**	**p-value**
Age (Years)	46.5 (12.4)	44.2 (12.3)	0.69
Sex	9F/2M	8F/1M	0.66
Race (Black/White)	0/11	3/6	**0.04**
Ethnicity (Hispanic-Latino/non-Hispanic-Latino)	2/9	1/8	0.66
Weight (kg)	95.2 (22.6)	100.9 (28.1)	0.62
BMI (kg/m^2)	33.8 (7.6)	34.4 (7.8)	0.86
Systolic blood pressure (mmHg)	132 (13.0)	123 (13)	0.14
Diastolic blood pressure (mmHg)	85 (4)	79 (8)	**0.04**
Eating window (hours)	15.2 (0.7)	15.5 (1.1)	0.47
Fasting glucose (mg/dL)	95 (10)	95 (13)	0.92
Fasting insulin (mU/L)	11 (6)	10 (5)	0.84
Hemoglobin A1c (%)	5.4 (0.4)	5.6 (0.4)	0.46
2 hour OGTT glucose (mg/dL)	142 (45)	96 (24)	**0.01**
HDL (mg/dL)	50 (14)	60 (18)	0.19
Triglycerides (mg/dL)	144 (54)	87 (21)	**0.01**
LDL (mg/dL)	95 (24)	105 (19)	0.31
TSH (mU/L)	2.2 (0.8)	1.4 (0.6)	**0.01**

[a] As the current paper is a secondary analysis, the baseline characteristics are summarized in Supplemental Table 2 for reader convenience. The full results were previously reported in Chow, L.S., et al., *Time-Restricted Eating Effects on Body Composition and Metabolic Measures in Humans who are Overweight: A Feasibility Study.* Obesity (Silver Spring), 2020. **28**(5): p. 860-869. [b] Continuous measures are reported as mean (SD). Categorical measures are reported as counts.

Supplemental Table S3: Correlation between weight loss and change in eating occasion, food types and beverages

	**TRE (*n* = 11)**		**Non-TRE (*n* = 9)**	
	Absolute weight loss correlation coefficient *p*-value	Percent weight loss correlation coefficient *p*-value	Absolute weight loss correlation coefficient *p*=value	Percent weight loss correlation coefficient *p*-value
Eating occasion*	0.009<br>0.98	0.068<br>0.84	0.269<br>0.48	0.375<br>0.32
**Food types***				
Complete meal	0.441<br>0.17	0.579<br>0.06	-0.080<br>0.84	0.043<br>0.91
Incomplete meal	-0.425<br>0.19	-0.382<br>0.25	-0.026<br>0.95	-0.147<br>0.71
Less balanced meal	0.042<br>0.90	-0.082<br>0.81	0.067<br>0.86	-0.006<br>0.99
Vegetarian meal	-0.384<br>0.24	-0.377<br>0.25	-0.183<br>0.64	-0.243<br>0.53
High quality snack	0.526<br>0.10	0.341<br>0.31	0.567<br>0.11	0.670<br>**0.05**
Mixed quality snack	-0.602<br>**0.05**	-0.595<br>**0.05**	0.289<br>0.45	0.275<br>0.47
Low quality snack	-0.226<br>0.50	-0.118<br>0.73	0.087<br>0.82	0.184<br>0.63
**Beverages***				
Water	0.279<br>0.41	0.240<br>0.48	-0.088<br>0.82	0.020<br>0.96
Caffeinated	-0.246<br>0.47	-0.112<br>0.74	0.420<br>0.26	0.440<br>0.24
Sugary	-0.379<br>0.25	-0.436<br>0.18	0.482<br>0.19	0.437<br>0.24
Artificially sweetened	-0.090<br>0.79	-0.139<br>0.68	-0.200<br>0.61	-0.169<br>0.66
Dairy	-0.315<br>0.35	0.037<br>0.91	-0.116<br>0.77	0.006<br>0.99
Alcohol	-0.008<br>0.98	0.110<br>0.75	0.117<br>0.76	0.055<br>0.89

*Change in eating occasions, food types and beverages were calculated as the difference between T2 (end-intervention) and T0 (baseline). Pearson correlation coefficient was calculated by treatment group to evaluate the correlation between weight loss and change in eating frequency, food types and beverages
